# Comparison of wrist actimetry variables of paretic upper limb use in post stroke patients for ecological monitoring

**DOI:** 10.1186/s12984-023-01167-y

**Published:** 2023-04-27

**Authors:** Gilles Dusfour, Denis Mottet, Makii Muthalib, Isabelle Laffont, Karima Bakhti

**Affiliations:** 1 CARTIGEN, University Hospital of Montpellier, Montpellier, France; 2 EuroMov Digital Health in Motion, Univ Montpellier, IMT Mines Alès, Montpellier, France; 3 Physical and Rehabilitation Medicine, Montpellier University Hospital (CHU), Montpellier, France

**Keywords:** Actimetry, Stroke, Upper limb hemiparesis, Actimetric variables

## Abstract

**Background:**

To date, many wrist actimetric variables dedicated to measuring the upper limbs (UL) in post-stroke patients have been developed but very few comparisons have been made between them. The objective of this study was to compare different actimetric variables of the ULs between a stroke and healthy population.

**Methods:**

Accelerometers were worn continuously for a period of 7 days on both wrists of 19 post-stroke hemiparetic patients as well as 11 healthy subjects. Various wrist actimetry variables were calculated, including the Jerk ratio 50 (JR50, cumulative probability that the Jerk Ratio is between 1 and 2), absolute (FuncUse30) and relative (FuncUseRatio30) amounts of functional use of movements of the ULs with angular amplitude greater than 30°, and absolute (UH) and relative (UseHoursRatio) use hours.

**Results:**

FuncUse30, FuncUseRatio30, UH, UseHoursRatio and JR50 of the paretic UL of stroke patients were significantly lower than in the non-dominant UL of healthy subjects. Comparing the ratio variables in stroke patients, FuncUseRatio30 was significantly lower than UseHoursRatio and JR50, suggesting a more clinically sensitive variable to monitor. In an exploratory analysis, FuncUseRatio tends to decrease with angular range of motion for stroke patients while it remains stable and close to 1 for healthy subjects. UseHoursRatio, FuncUseRatio30 and JR50 show linear correlation with Fugl-Meyer score (FM), with r^2^ equal to 0.53, 0.35 and 0.21, respectively.

**Conclusion:**

This study determined that the FuncUseRatio30 variable provides the most sensitive clinical biomarker of paretic UL use in post-stroke patients, and that FuncUseHours—angular range of motion relationship allows the identification of the UL behaviour of each patient. This ecological information on the level of functional use of the paretic UL can be used to improve follow-up and develop patient-specific therapy.

**Supplementary Information:**

The online version contains supplementary material available at 10.1186/s12984-023-01167-y.

## Background

Stroke is one of the leading causes of disability worldwide, with a global prevalence rate that has been increasing over the past 30 years [1]. Despite the accumulated research on rehabilitation of the paretic upper limb (UL) following a stroke, a large majority of patients continue to present non-use of paretic UL at the chronic stage which impacts their quality of daily life [2]. Only 5 to 20% of stroke survivors regain sufficient paretic UL function after 6 months [3], which leaves the majority of chronic post stroke patients unable to use their paretic UL in their daily life.

Current methods of quantifying movement of the UL rely primarily on clinical deficit scores such as the Fugl-Meyer (FM) test [4], or on more functional tests like the Wolf Motor Function Test (WMFT), Action Research Arm Test (ARAT) or questionnaires (Motor Activity Log—MAL). More recent work focused on the direct visual observation of stroke patients ULs by hospital practitioners in a clinical environment during 7 days [5]. This work found that the ratio of use activity between the paretic limb and the non-paretic limb is around 0.69 for stroke patients [5] whereas it is 0.95 for healthy subjects (non-dominant/dominant) [Bailey et al., 2014]. The human assessor method used by McLaren [5] has the advantage of identifying with certainty the periods of functional use of the UL as assessed directly by the clinician. However, the time and human resource costs of performing these measurements reduce its applicability to monitor multiple patients, and moreover, limiting observations in a clinical setting might not reflect real life of patients in a home environment.

Alternatively, a commonly used quantitative and objective technique to quantify functional UL movements relies on methods based on actimeters or gyroscopes [Bailey et al., 2014] positioned on the two wrists over a period of time ranging from 2 to 7 days. The functional UL movement results of Bailey's work [6, 7] are based on the calculation of activity counts directly from the acceleration signals originally developed by Uswatte [8]. Bailey derived other variables from the accelerometric measurements, such as use hours based on acceleration thresholds and median bilateral magnitude based on the magnitude of the accelerations measured at each wrist. The correlations of UL activity count with clinical scores such as the FM or the WMFT showed high variability between studies [6, 7]. Although Lang et al. [9] showed a strong correlation (r^2^ = 0.62) between the use hours and the WMFT, a more recent study shows a weaker correlation between the median bilateral magnitude and the FM Score (r^2^ = 0.32) or the WMFT score (r^2^ = 0.34) [10]. Recently, Pan et al. [11] developed new accelerometric variables based on the Jerk, which is the derivative of acceleration. Pan et al. [11] showed that the Jerk ratio (JR) has a very high sensitivity to the amount of UL motion as well as a very high correlation with the median bilateral magnitude. Leuenberger et al. [12] extended the method by using inertial sensors (i.e., accelerometer and gyroscope) to separate functional vs. non-functional UL movements. A functional UL movement occurs when the forearm is oriented horizontally (± 30°), which is usually the case in UL manipulation activities. Leuenberger et al. [12] found excellent correlation of the functional use ratio (FuncUseRatio30) with the box and block (BB) test (r^2^ = 0.9). Lum et al. [13] chose to synchronise accelerometers with video recordings of healthy and hemiparetic subjects performing activities of daily life. The video recordings were used both to accurately measure the amount of functional UL movement in the laboratory over a given period of time and to serve as a basis for labelling actimetric data as functional, non-functional and unknown movement. This labelling was then used to develop several machine learning algorithms to separate functional from non-functional UL movements. Although the activity counts showed low correlation with the video results (r^2^ = 0.57), the machine learning algorithms showed excellent results (r^2^ = 0.81).

The majority of studies on wrist actimetric monitoring of post-stroke patients monitor the quantity of UL movement and its bilateral ratio. While these indicators seem relevant for measuring imbalances between the UL motion, it remains difficult to draw conclusions about the functional imbalance and the physical capacities and limitations of the patients' UL in their ecological environments. Leuenberger's work provides a significant advance by identifying the amount of movement around the horizontal plane with an amplitude of ± 30°. For the first time, it is possible to easily discriminate a "functional" amount of movement in an ecological environment. However, Leuemberger's work required inertial sensors that are relatively expensive and have little energy autonomy. Finally, more recent studies based on artificial intelligence algorithms show promising results in identifying functional motion but require a significant amount of time for manual classification of motion. Moreover, these algorithms do not yet distinguish between movements of different amplitudes. This makes it difficult to identify the physical capabilities and limitations of post-stroke subjects in their ecological environments while it is necessary to turn accelerometer data into clinical meaningful data [14].

In this study we recorded 3D acceleration at each wrist, over a period of 7 days, in the volunteers’ home (ecological) environment. We then adapted and compared different accelerometric variables (UseHours, UseHoursRatio, FuncUse30, FuncUseRatio30 and JR50) between a population of 19 stroke patients and 11 healthy subjects to determine the actimetric variable that has the greatest sensitivity to stroke hemiparesis-related upper limb deficits in order to guide clinical decision making.

## Methods

### Participants

Each participant was asked to sign an informed consent form approved by the Institutional Review Board (the local ethics commission). Patients were recruited in the PRM unit between December 2019 and May 2021. The post-stroke participants met the following inclusion criteria: (1) diagnostic criteria for stroke, (2) people after an ischemic or haemorrhagic stroke that suffered from a moderate to mild paretic arm (defined as a Fugl Meyer—Upper Extremity—FM-UE score > 15/66), in the chronic stage of recovery (> 6 month post-stroke). (2) 18 years or older. The exclusion criteria were the following: (1) Mini-Mental Status Examination score < 24 [15], (2) strong neglect with a Bell’s test > 15 bells (3) orthopaedic or rheumatologic injury of their upper limb, (3) pregnancy*.* The controls had no self-reported injuries that would alter or impair their use of either UL.

### Procedures

Accelerometers (Axivity Ax3, Newcastle upon Tyme, UK) were placed on each wrist for all participants. The participants were asked to wear the accelerometers for 7 days without removing them. Data acquisition was performed at a frequency of 50 Hz coupled with a cut off of 8 g for the measurement of acceleration in the three spatial directions. At this acquisition rate, the sensors are capable of recording data for 14 days. The subjects were asked to keep the sensors on day and night even while showering. The accelerometers were recovered at the end of the 7 days to extract the data using the OmGui software provided by Axivity. The data were sliced day by day to obtain daily acceleration data values over the whole day. The data were then saved in csv format so they can be read by any programming language.

### Data Analyses and calculation of variables

Data processing was done using the *python 3.7* programming language. The *numpy* and *scipy* libraries are notably used for numerical calculation operations (derivation, frequency analysis). The *scipy* library allows the application of a low pass filter with a cut-off frequency of 10 Hz in order to remove noise. The magnitude of the acceleration vector (EN: Euclidean Norm) is then calculated for each time step of the two actimeters (via the acceleration data at a given time t: a_x_(t); a_y_(t); a_z_(t)).

1 $$\begin{array}{c}EN\left(\mathrm{t}\right)=\sqrt{{\mathrm{a}}_{\mathrm{x}}^{2}+{\mathrm{a}}_{\mathrm{y}}^{2}+{\mathrm{a}}_{\mathrm{z}}^{2}} .\end{array}$$

### Jerk

The time derivative of the acceleration at a given time t allows us to obtain the Jerk, noted J, in the three directions of space via the following calculation (finite difference centred approximation):

2 $$\begin{array}{c}{\mathrm{J}}_{\mathrm{i}}\left(\mathrm{t}\right)=\frac{{\mathrm{a}}_{\mathrm{i}}\left(\mathrm{t}+\mathrm{dt}\right) - {\mathrm{a}}_{\mathrm{i}}\left(\mathrm{t}-\mathrm{dt}\right)}{2\mathrm{dt}},\end{array}$$

where *i* represents the three directions of space *x*, *y* and *z*, *a* is the scalar value of the acceleration and dt the sampling time step (i.e. 50 Hz). Physically, the Jerk represents the rate of change of the acceleration vector. It is then possible to calculate the magnitude of the Jerk:

3 $$\begin{array}{c}Jer{k}_{Mag}=\sqrt{{J}_{x}^{2}\left(t\right)+{J}_{y}^{2}\left(t\right)+{J}_{z}^{2}\left(t\right)}.\end{array}$$

Pan et al., [Pan et al. 2020] showed that the jerk ratio (JR) is sensitive to the degree of UL mobility. In our study, the JR is defined as the ratio of the jerk amplitude of the paretic (non-dominant) limb to the sum of the nonparetic (dominant) limb and paretic limb jerk:

4 $$\begin{array}{c}Jer{k}_{ Ratio}=2x\frac{\left|Jer{k}_{paretic}\right|}{\left|Jer{k}_{paretic}\right|+\left|Jer{k}_{non-paretic}\right|}.\end{array}$$

We have adapted the JR formula from Pan et al. [11] so that it is comparable to the use hours and functional movement ratios (see details in the following sections). With the objective that a JR equal to 1 means an equal contribution from both ULs. Points where both the jerk of the paretic or non-paretic side is equal to zero are excluded from the study. It is then possible to calculate the histogram and density function of the JR for each measurement day. The density function is normalised to give a total distribution of 2. Such a normalisation is chosen in order to extract a representative variable, the JR50 [11], comparable to all ratio variables in this article. The JR50 was calculated as the area under the curve when the JR is between 1 and 2. A JR50 higher than 1 means a preponderant use of the paretic (non-dominant) arm while a JR50 of less than 1 means a preponderant use of the non-paretic (dominant) arm.

### Functional movement

In quasi-static conditions, the calculation of the angle of elevation of the forearm with respect to the gravity vector takes the form of Eq. (5), following the trigonometric laws [Fisher, C. J. (2010).]:

5 $$\begin{array}{c}\alpha \left(t\right)=arcos\left(\frac{{a}_{y}\left(t\right)}{EN\left(t\right)}\right).\end{array}$$

We have extended this method for dynamic conditions, without any additional calculation, as preliminary results have shown excellent prediction of the elevation angle regardless of the dynamics of the movements with an accelerometer and Eq. (1) (see Additional file 1). Leuenberger et al. [12] estimates that the ULs perform a functional movement when there is a variation in the angle of inclination of the arm greater than 30° and that this same angle of inclination is between ± 30° around the horizontal (to avoid data from walking) all within non-overlapping time window of 0.5 s. The mathematical formulation is as follows:

6 $$\begin{array}{c}\left|\mathrm{\alpha }\right|\le 30^\circ and {\mathrm{\alpha }}_{max}-{\alpha }_{min}\ge 30^\circ .\#\end{array}$$

The formulation of this hypothesis is motivated by the fact that the majority of everyday movements take place in the sagittal plane [17] and mainly above the hip [18]. A functional movement iteration counter is created for both upper limbs for each day. The counter, the FuncUse30 (Functional Use for range of motion greater than 30°), is updated for each functional movement detected. The absolute values of FuncUse30 and its ratio (FuncUseRatio30, paretic/non-paretic or non-dominant/dominant) are presented as a boxplot with the median value of the 7 days of measurements.

“The idea that a functional movement must have sufficient amplitude makes sense, but the choice of an amplitude of 30° in Eq. (6) is arbitrary [12]. Here, we explored how changing this amplitude also changes the number of functional movements. We defined 9 movement amplitudes from 10 to 90 degrees, in 10-degree steps (i.e., [0°–10°]...[80°–90°]). We then counted the number of functional movements for each amplitude.”

### Use hours

The calculation of use hours follows the formulation presented by Waddell et al. [19] and Bailey et al. [6]. In order to use signal frequencies of 30 Hz comparable to the literature, the initial 50 Hz signal was downsampled to 30 Hz using the "decimate" function of the *scipy* library of the python programming language [6, 19]. Next, the data were bandpass filtered between 0.25 and 2.5 Hz, and then converted to activity counts (0.001664 g/count) on non-overlapping 1 s windows. The number of hours of use (UH) corresponds to the total amount of time the UL is assumed to be active. This variable is calculated by adding all seconds where the activity count is greater than 2. It is then possible to define use hours ratio (UseHoursRatio) between the paretic (non-dominant) and non-paretic (dominant) UL.

### Statistical analysis

The statistical analysis was performed with the programming language *python 3.8,* using *scipy* and *pandas* packages. The criterion for a significant difference was p < 0.05. All statistical analyses were performed at 95% confidence. Nonparametric tests had to be applied because normality tests (Shapiro–Wilk) showed that some of the data groups do not have normal distribution. For each type of variable (JR50, UH, UseHoursRatio, FuncUse30, FuncUseRatio30), a value was calculated for each subject and for each day. This corresponds to 7 values per subject and per variable. For each subject and each variable, the median value of the 7 days of measurements was saved and stored. The visual representations of the variables from the accelerometric data were examined using boxplots for each population. The values shown as dots in the boxplot represent the median of the 7 days of measurements per patient. The boxplots then show the median and interquartile range of the healthy and stroke populations. The absolute values (FuncUse30, UH) of the healthy population were statistically compared with those of the stroke population via non-parametric Mann–Whitney tests to identify differences in behaviour between the two independent populations. The ratios (FuncUse30R, UseHoursRatio, JerkRatio) were compared between the two populations and within the stroke population to identify differences in response between the populations and between the variables. Again, a non-parametric Mann–Whitney test was applied with a Bonferroni correction for multiple comparisons for independent data (healthy vs stroke) and a Wilcoxon test was applied with a Bonferroni correction for paired data (difference between FuncUse30, FuncUseRatio30, UH, UseHoursRatio and JR50 in the stroke population).

## Results

### Patients

In this study, 11 healthy (7 women) and 19 post-stroke patients (8 women) participated. The characteristics of the stroke patients and healthy subjects are summarized in Table 1. The average FM-UE score of the hemiparetic subjects was 50.5 [27–66]. Only six patients had a moderate FM score (range 15–35), the other thirteen patients had a mild score (range > 35). The results of all actimetric variables are summarised in Table 2.

**Table 1:** Characteristics of stroke patients and healthy subjects

	Post stroke patients	Healthy volunteers
Number	19	11
Age in years	67 ± 12 [47–83]	58 ± 20 [18–75]
Gender	11 males, 8 females	4 males–7 females
Affected body side	11 right, 8 left	–
Dominant side affected	8 (42%)	–
FM-UE Score (/66)	45.6 ± 16 [27–66]	–

**Table 2:** Summary of all absolute and ratio variables for the stroke and healthy subjects

	Stroke	Healthy	p
UH Paretic (non-dominant)	5.34 h	8.13 h	0.0049**
UH Non Paretic (dominant)	7.56 h	9.15 h	0.53
FuncUse30 Paretic (ND)	831 movements	4040 movements	0.0011**
FuncUse30 Non Paretic (D)	4083 movements	4924 movements	1
JR 50	0.78	0.93	0.068
FuncUseRatio30	0.18	0.78	0.0052**
UseHoursRatio	0.6	0.94	0.017*

*p < 0.05

**p < 0.01

***p < 0.001

### Jerk ratio

**Fig. 1:**
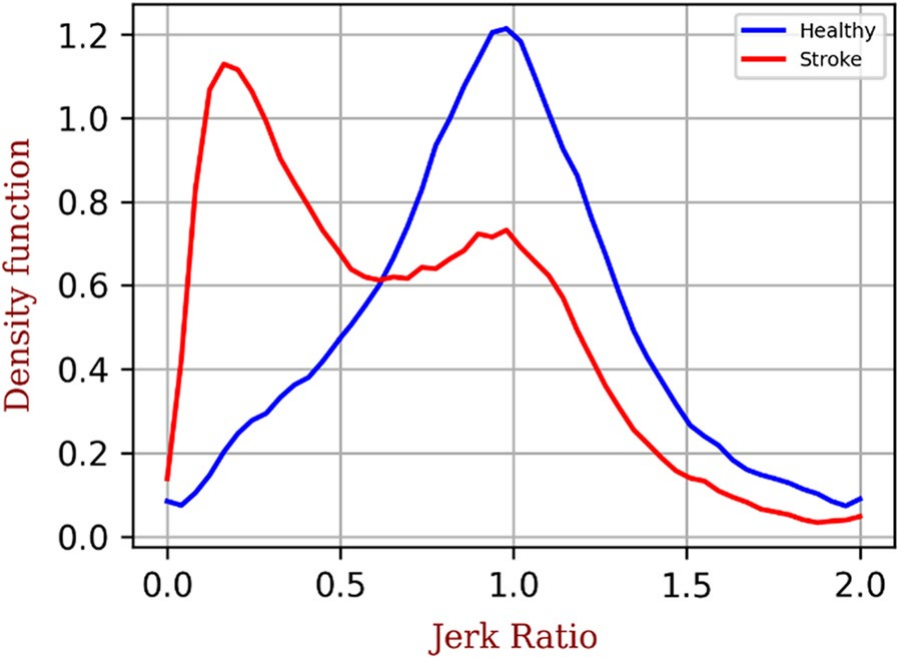
Comparison of the jerk ratio (JR) normalised density function of a stroke (in red) and healthy (in blue) subject. A JR of 0 indicates use of the non-paretic (dominant) limb and a ratio of 2 indicates use of the paretic (non-dominant) limb. The healthy subject has a maximum density for a JR of 1 (use of both limbs at the same time) while the maximum density of the JR for the stroke patient is 0.2 (predominant use of the non-paretic limb)

Figure 1 shows the histogram and density function (DF) of the JR for a healthy subject and a stroke patient on a representative day. We can see that the maximum density function histogram for the healthy subject is centred on a JR value of 1, which highlights a balance in the movement of the upper limbs. A slight peak can also be seen at a JR value of 0 and 2, highlighting a non-negligible amount of probability of movement of the dominant limb only or non-dominant limb only, respectively. For the stroke patient, it can be seen that the maximum DF is positioned at a JR value of 0.2, highlighting a preponderance of movement of the non-paretic limb.

### Absolute use hours, functional movements and variables ratios

**Fig. 2:**
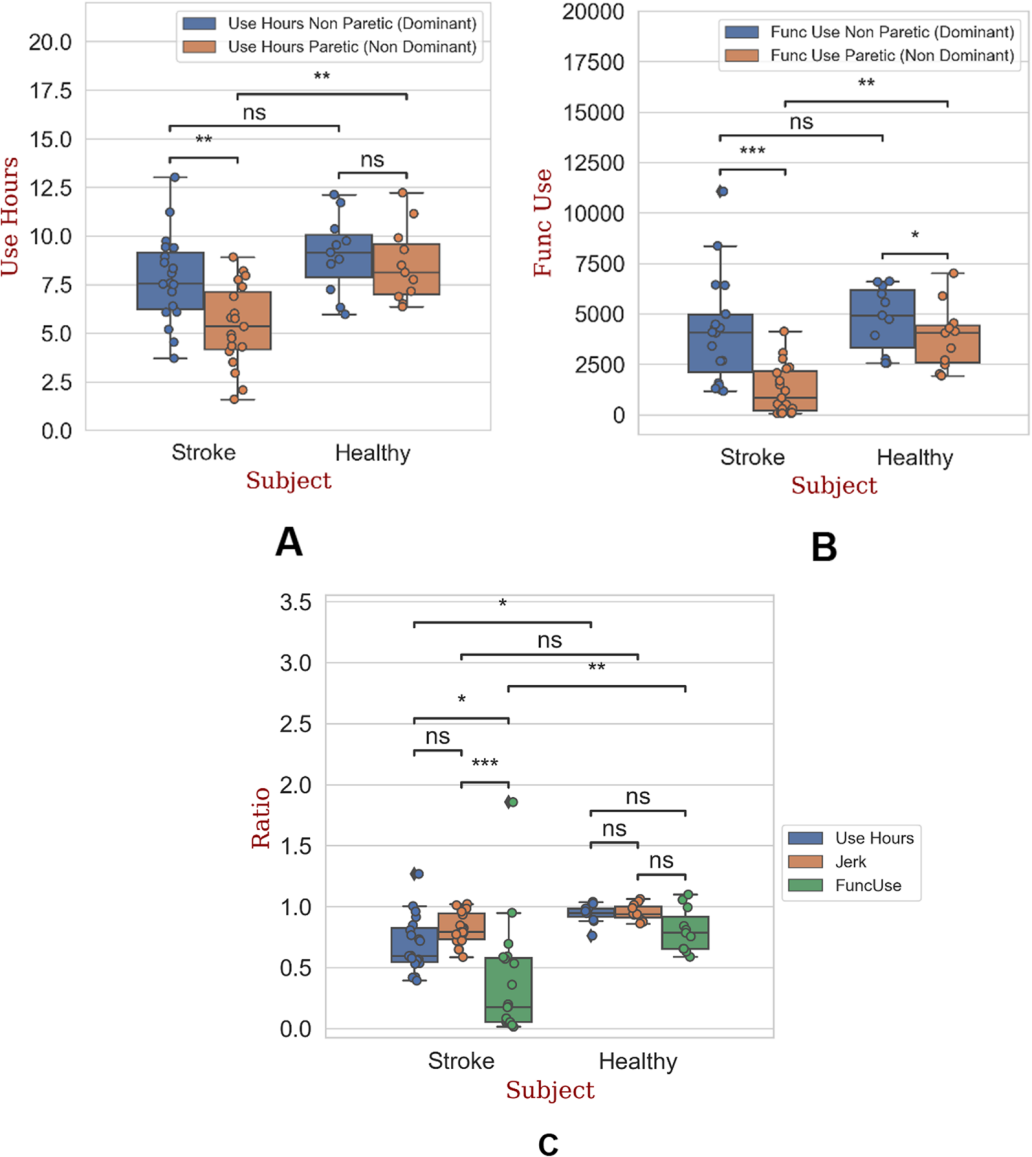
**A** Boxplot of absolute use hours for the non-paretic (dominant) and paretic (non-dominant) upper limbs of the stroke and healthy subjects. **B** Boxplot of absolute functional movement (FuncUse30) for the non-paretic (dominant) and paretic (non-dominant) upper limbs of the stroke and healthy subjects. **C** Boxplot of all ratio variables (JR50, FuncUseRatio30, UseHoursRatio), for the stroke and healthy subjects. *Ns* non-significant, *p < 0.05, **p < 0.01, ***p < 0.001. Dots represents the median value of the 7-days for each subject. Diamond represents outliers

The UH and FuncUse30 results are given as boxplots in Fig. 2A, B, respectively. In both groups, the UH of the non-paretic (dominant) limbs are greater than the UH of the paretic (non-dominant) limbs. The UseHours of the dominant (non-paretic) limbs of the healthy and stroke subjects are 1.12 and 1.5 times greater than their non-dominant (paretic) limbs, respectively. While there was no significant difference (p > 0.05) in the upper limb UseHours for the healthy population, the stroke population significantly spent more time using their non-paretic upper limb. Moreover, the UseHours of the non-dominant limbs of the healthy subjects were significantly 1.5 times greater than those of the stroke subjects (p < 0.01).

In an identical manner, the FuncUse30 of the paretic limbs are 5 times lower than the FuncUse30s of the non-paretic limbs (831 movements vs 4083 movements, p < 0.001). Similarly, to UH, the FuncUse30s of the non-dominant limbs of healthy subjects are 4.86 times higher than the FuncUse30s of the paretic limbs of pathological subjects (831 movements vs 4040 movements p < 0.01).

Figure 2C shows the boxplots of the median ratio (i.e., dominant/non-dominant) for the UseHoursRatio, JR50 and the FuncUseRatio30. The median UseHoursRatio and FuncUseRatio over 7 days of measurement was significantly lower (WMW test: p < 0.05 for UseHoursRatio and p < 0.01 for FUR) for the stroke (UseHoursRatio: 0. 0.6, JR50: 0.78 and FUR: 0.18) than for the healthy (UseHoursRatio: 0.94, JR50: 0.93 and FUR: 0.78) population. Finally, in the stroke population, the FUR is significantly lower than the JR50 and UseHoursRatio (respectively p < 0.001 and p < 0.05). The ratios do not present significant differences in the healthy population

### Functional movements in relation to movement amplitude

**Fig. 3:**
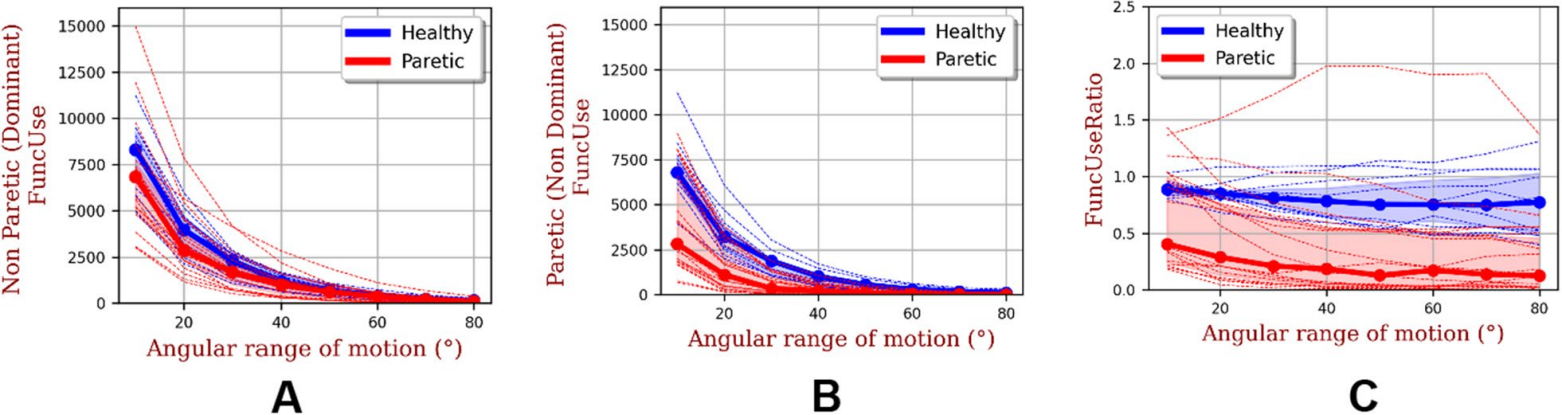
**A**–**C** plot of absolute functional movement for non-paretic (dominant) and paretic (non-dominant) and functional movement ratio (FuncUseRatio) in relation to movement amplitude for the stroke (in red) and healthy (in blue) subjects. The dotted-curves represent the median values of each population, the filled areas around the median values represent the interquartile range (q25–q75). The dashed curves represent each subject

Figure 3A–C show the FuncUse's and the FuncUseRatio’s in relation to the range of motion of the dominant limb (non-paretic), non-dominant limb (paretic) and the of each of the subjects observed in this study as well as the median amplitudes for each population. FuncUse's are close to 0 for angular amplitudes of [80°–90°] for both populations. The median values of the two populations for FuncUse and FuncUseRatio are represent in dotted lines. While the FuncUse on the non-paretic (dominant) side is relatively equivalent for both populations and present no statistical differences, it is much lower on the paretic (non-dominant) side for all angular amplitudes and present statistical differences for angular range of motion greater than 20° (p < 0.05 for [20°–30°] interval and p < 0.01 for angular amplitude greater than 30°). Finally, it can be seen in Fig. 3C that the median FuncUseRatio remains relatively constant and close to 0.8 for the healthy population with increasing angular amplitude whereas it decreases sharply for the hemiparetic population (from 0.4 to 0.2). Like absolute values, the median FuncUseRatio of the healthy population is significantly greater than that of the stroke population for angular amplitudes greater than 20° (p < 0.05 for [20°–30°] interval and p < 0.01 for angular amplitude greater than 30°)

**Fig. 4:**
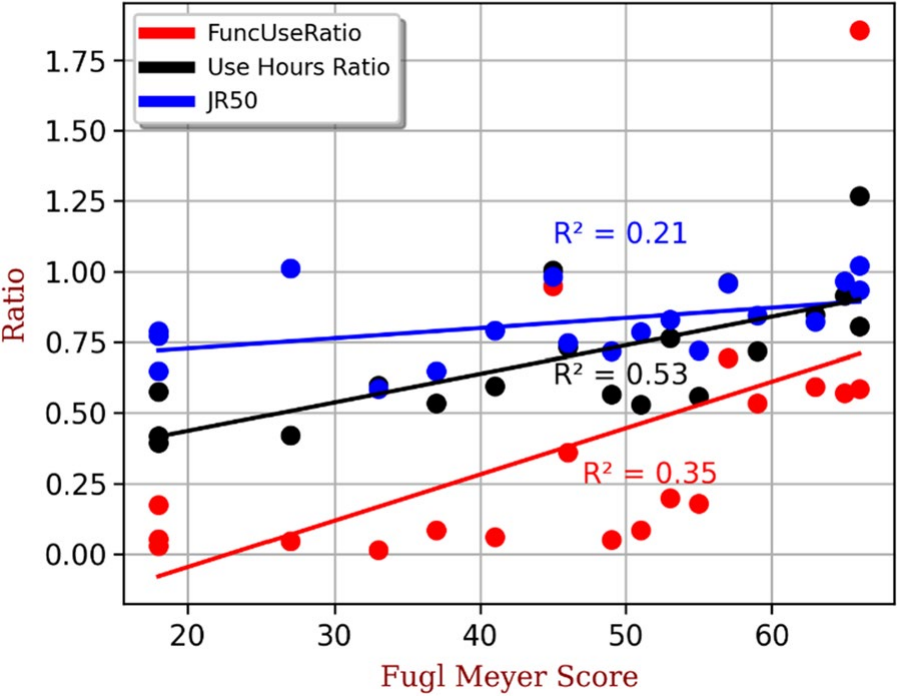
Relationship between FuncUseRatio30, UseHoursRatio and JR50 with Fugl Meyer Score. Each dot represents one stroke subject

### Relationship between relative variables and Fugl Meyer Score

Figure 4 shows the scatter plot of the relationships of FuncUseRatio30, UseHoursRatio and JR50 with the FM score. All three ratios tend to increase with the FM score. The UseHoursRatio had the highest correlation to the FM (r^2^ = 0.53), followed by the FuncUseRatio30 (r^2^ = 0.35) and the JR50 (r^2^ = 0.21). The JR50 had a really small slope (0.0036), while the UseHoursRatio and FuncUseRatio30 had a much larger slope (0.01 and 0.016, respectively).

## Discussion

The aim of the study was to compare different wrist actimetry variables between stroke and healthy volunteers over a 7 day period in their home environment to determine which actimetry variables have the greatest sensitivity to paretic UL functional use and can be used to guide therapist’s decision making. We performed, to our knowledge, the first study in stroke patients that calculated over an extended 7-days period FuncUseRatio, UseHoursRatio and JR50 variables via two simple and lightweight wrists worn accelerometers, and compared these values with values acquired in a healthy population. Accordingly, we derived new actimetry variables, in particular, we were able to calculate average FuncUse and FuncUseRatio over a large range of elevation angles.

Previous studies have measured the amount of functional movement of the UL (FuncUse) in an ecological environment via IMUs placed at the wrist for a period of only 48 h [12]. The arm elevation was calculated using the same accelerometric metrics to which the authors added the calculation of the yaw angle to identify movements in the horizontal plane. In our study, we chose to use actimeters with a battery autonomy of more than one week for an acquisition frequency of 50 Hz and thus to be more representative of the patient's ecological behaviour in the home environment. It is noted that [12] demonstrated a linear relationship between the Box and Blocks Test and the ratio of movement of the paretic limb to the non-paretic limb.

Our work builds on [12], by exploring well known variables like the UseHoursRatio and more recent variables like the JR50 and by performing a comparison with a healthy population. The UseHours values and UseHoursRatio calculated in our study are equivalent to those of Bailey and Waddell [6, 19]. Like the study of Bailey et al. [6], we showed significantly greater UseHours and UseHoursRatio of the non-dominant limb of the healthy subjects compared to the paretic limbs of the stroke patients as well as a significantly greater FuncUseRatio30 in the healthy subjects than in the stroke patients. Futhermore, we confirmed a significantly greater FuncUseRatio30 in the healthy subject than in the stroke patients, where healthy subjects show on average three times more daily movement of the non-dominant limb than stroke subjects using the paretic limb. Indeed, healthy subjects performed approximately 4000 functional movements per day with their non-dominant limb whereas post-stroke patients realized only 800 movements per day with their paretic limb. Moreover, the healthy subjects show a FuncUseRatio30 close to 0.8, meaning an almost equal use of the dominant and non-dominant upper limbs while the stroke patients show a very low median FuncUseRatio30 close to 0.2, which indicates 30 functional movements of the non-paretic limb for one functional movement of the paretic limb. However, patients show an equivalent amount of functional movements of the non-paretic limbs to that of the dominant limb of the healthy subjects. This suggests that the stroke patients studied here maintain a relatively normal amount of non-paretic UL movements.

The JR50 reflects the ratio of the amount of movement in a given time frame between the two limbs. While this ratio is balanced in healthy subjects, it shows a slight imbalance in stroke subjects. These results show that stroke patients perform less movement, both functional and non-functional, with their paretic limb than with their non-paretic limb when compared with the healthy population. Figure 4 shows the increased sensitivity of the FuncUseRatio30 to the Fugl Meyer scores compared to the other two variables, JR50 and UseHoursRatio. Indeed, the slope of the linear relationship of the FuncUseRatio30 with the FM score is 4.6 and 1.6 times greater than for the JR50-FM and the UseHoursRatio-FM relationships, respectively. In addition, we observed a slight non-linear behaviour of the FuncUseRatio-FM relationship. Indeed, the FuncUseRatio increases strongly for FM scores above 50, whereas it remains relatively low and constant for values below 50.

To define a functional upper extremity movement, we selected a limit of ± 30° from the horizontal for the forearm elevation angle [12]. This choice of 30° might be disputable, for example, because a large proportion of stroke patients show uncontrolled flexion of the healthy elbow when walking. This phenomenon is called "associated reaction" and may have an influence on the results of our study [20]. To better understand the sensitivity of functional use to variations in limits for forearm angle, we decided to explore the evolution of FuncUseRatio and FuncUse as a function of forearm angle limits. For both populations, the functional use of the upper limbs decreases when the forearm angle limits increase. For the healthy population, the FuncUse of the non-dominant limbs is equivalent to that of the dominant limbs whatever the forearm angle limits. The FuncUse of the paretic limbs is always lower than that of the non-dominant limbs of healthy subjects for angular amplitudes greater than 20°. Our study highlights threshold values of forearm angle limits for which stroke patients strongly decrease the functional use of paretic limbs. The FuncUseRatio remains stable and close to 1 whatever the amplitude of the movements for the healthy subjects. The FuncUseRatio strongly decreases when the forearm angle limits increase for the hemiparetic subjects and this even for subjects who present a very good FM score. When analysing the FuncUseRatio curves as a function of the forearm angle limits, we observe different patient profiles. Some patients have FuncUseRatios very close to 1 for small angular amplitudes while others have very low FURs even for small forearm angle limits. In general, the study of FuncUse and FuncUseRatio in relation to forearm angle limits allows a better appreciation of the physical capacities of stroke subjects in their home environments.

Our work shows that actimetric scores are potentially clinically useful for grading targeted rehabilitation and monitoring UL recovery in stroke patients. Throughout the course of treatment, actimetric outcomes could complement conventional clinical assessments (paretic UL use) to monitor upper limb recovery after stroke and better evaluate the effectiveness of treatment.

This easy-to-perform actimetric protocol has been shown to be feasible for objectively measuring the use of the paretic UL in ADL. This measurement can be performed outside the clinic, in the patient’s own environment. FuncUseRatio30 indicates an amount of functional use of upper limb movements around the horizontal plane with an angular amplitude greater than 30°. FuncUseRatio30 is low (low use of paretic upper limb compared to non-paretic upper limb) in stroke patients (0.2). The FuncUseRatio30 increases with recovery of UL use, to near 0.8 (almost equal use of UL). The FuncUseRatio30 adds value as clinical assessments record the actual deficit or remaining ability of the stroke patient, but it is difficult to objectively know the actual functional use of the paretic upper limb in ADL. Sometimes stroke patients with a mild deficit (FM-UE above 40/66) do not use their paretic UL as they should. Yet, it is well known that non-use of the paretic UL limits recovery. A FuncUseRatio30 of less than 0.8 (with a FU of less than 4000) may direct rehabilitation towards paretic UL force use (i.e. movement constraint induced therapy).

The FuncUseRatio is complementary to clinical assessments when analysed with different angular amplitudes. In fact, stroke patients, even with a mild deficit, have a FuncUseRatio of less than 0.8–1 at high angular amplitude. Knowing the angular amplitude threshold (FuncUseRatio less than 0.8–1) allows us to guide rehabilitation towards intensifying the use of the paretic UL at a certain angular amplitude (individualized rehabilitation). In this study, we were able to provide a stratification of stroke patients:

- Patients with a FM-UE ≤ 20/66: the actimetry does not seem to be relevant for severe patients (Turolla et al., 2013) since these patients do not have the ability to move their UL.
- Patients with a FM-UE between 21 and 50/66: FuncUseRatio30 seems to be relevant for moderate to mild stroke patients with a threshold at FM-UE:50/66. These do not increase spontaneously the use of their paretic UL even if their FM-UE increases. These patients will need specific rehabilitation focused on the use of the paretic UL at home in ADLs. A change in FuncUseRatio30 will be significant and will show a recovery in the use of the paretic UL at home in ADLs.
- Patients with a FM-EU greater than 50: These patients appear to use the paretic UL as much as they increase their FM-UE.

Advances in accelerometric data processing and analysis will provide real-time information to stroke patients on the use of their paretic UL. In a short term, tele-rehabilitation support (telephone, Internet, etc.) could be developed to encourage the stroke patients to use the paretic UL when the use score falls below a threshold for use over a given period of time (threshold and duration specific to each patient).

In the medium to long term, coupled with monitoring by a connected accelerometric bracelet, a digital assistant could be used for simple feedback to the patient or for detailed analysis for therapists, in order to improve the individualisation of follow-up. Stroke patients will be able to assess their FuncUseRatio on a daily basis through an application that may motivate them to make greater use of their paretic UL.

Another perspective would be to mix experimental method tools based on actimetry and artificial intelligence to identify with more precision what kind of movements is performed by the patients [21]. This identification of the movement will allow to better identify the physical capacities of hemiparetic patients and thus to develop specific patient therapies. It would be also relevant to accurately assess the FU measurement capability via a video ground truth measurement in a similar way to Lum et al. [13]. Similarly, it would be relevant to investigate the intra-subject variability of the different actimetric variables in order to identify the minimum detectable difference. In addition, other actimetric variables could have been calculated from our data to refine the study. Two such variables include the quantification of physical activity via the ENMO (Euclidean Norm Minus One) variable [22] as well as the quantification of smoothness during a functional movement [23].

It is now necessary to carry out an in-depth clinical study to identify different patient patterns, by enlarging the number of patients we involve and by covering a larger panel of different patients. In view of the greater sensitivity of the FuncUseRatio to Fugl-Meyer scores, it would be appropriate to use this variable in the longitudinal assessment of patients undergoing therapies programs. We are thinking in particular of therapies based on virtual reality tools and transcranial stimulation [24]. Like the FuncUseRatio30 developed by Leuenberger et al. [12] correlates linearly with the BBT, we showed that the FuncUseRatio30, the UseHoursRatio and the JR50 correlate linearly with the Fugl Meyer score. It would be interesting to investigate the correlation of such variable with clinical variables like the BBT, or other clinical assessments of the UL function. Interestingly, the tools developed in this article should make it possible to identify stroke patients with excellent actimetric results. It would then be relevant to deepen the study by correlating actimetric and clinical variables with other variables identifying motivation, environmental factors, anxiety and depression [2]. Such studies would allow the identification of other paths for performance improvement.

## Conclusions

This study comparing post-stroke and healthy subjects over a 7 day period in their home environment found significant differences in the calculated actimetric variables between healthy and post-stroke subjects. Although the healthy subjects had an UL FuncUseRatio30 close to 0.8, the post-stroke subjects had a ratio of about 0.2, indicating greater use of the non-paretic than paretic UL. However, the post-stroke subjects do not seem to overuse their non-paretic limb to compensate for the loss of motor skills in the paretic limb. For the stroke patients, our ratio variable results showed greater differences between the paretic and non-paretic UL use with the FuncUseRatio30 than the JR50 and UseHoursRatio, suggesting that FuncUseRatio30 has greater sensitivity to characterise paretic UL use and be used as a biomarker for clinical decision making. Finally, our exploratory analysis of the relationship between FuncUseRatio and angle limits showed different patterns of behaviour of the patients' ULs. While half of the patients analysed show very low FUR (0.25) for small angle limits, the other half show FUR close to 1 for the same angle limits. It is now possible to discriminate with more precision the movements of the ULs of stroke subjects. Overall, the novel wrist accelerometer analysis and results of this study show the interest of using different variables for the longitudinal follow-up of stroke patients with UL hemiparesis and thus evaluate different rehabilitation therapies.

## Supplementary Information

**Additional file 1:** Inertial sensor vs accelerometer elevation angle estimation.

## Data Availability

The datasets used and/or analysed during the current study are available from the corresponding author on reasonable request.
