# Supplementary material for: Comparison of wrist actimetry variables of paretic upper limb use in post stroke patients for ecological monitoring
Source: J Neuroeng Rehabil. 2023 Apr 27;20:52. doi: 10.1186/s12984-023-01167-y (PMC10134627; doi:10.1186/s12984-023-01167-y)
Supplement: Supplementary file 1 — Additional file 1: Inertial sensor vs accelerometer elevation angle estimation. [file 12984_2023_1167_MOESM1_ESM.docx]

# Additional File 1: Inertial sensor vs accelerometer elevation angle estimation

In quasi-static conditions, the calculation of the angle of elevation of the forearm with respect to the gravity vector takes the form of equation 1, following the trigonometric laws [Fisher, C. J. (2010).]:

$$\begin{aligned} \alpha\left( t \right)=arcos\left( \frac{a_{y}\left( t \right)}{svm\left( t \right)} \right)\#\left( 1 \right) \end{aligned}$$

We have extended this method for dynamic conditions, without any additional calculation, as preliminary results have shown excellent prediction of the elevation angle regardless of the dynamics of the movements.

Figure S1 shows four comparisons of angle measurements obtained by an Xsens Dot inertial sensor (IMU) and an accelerometer using Equation 1 for functional movements. The root means square error (RMSE) between the two signals is given in the title of each sub-figure.

| 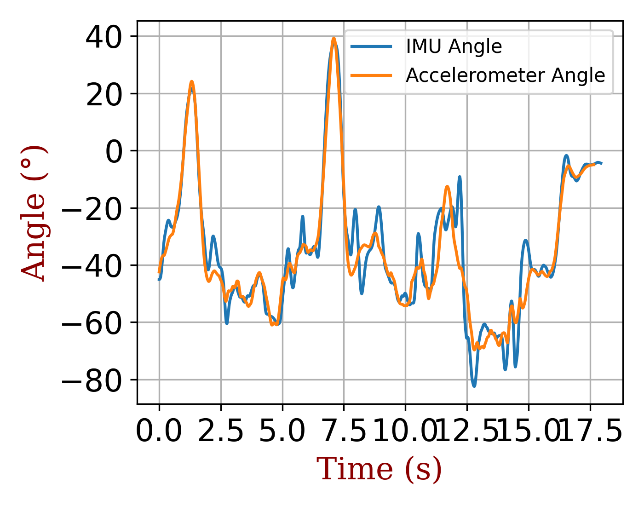  FigS1.1 Reaching movement 1. RMSE: 6.9° | 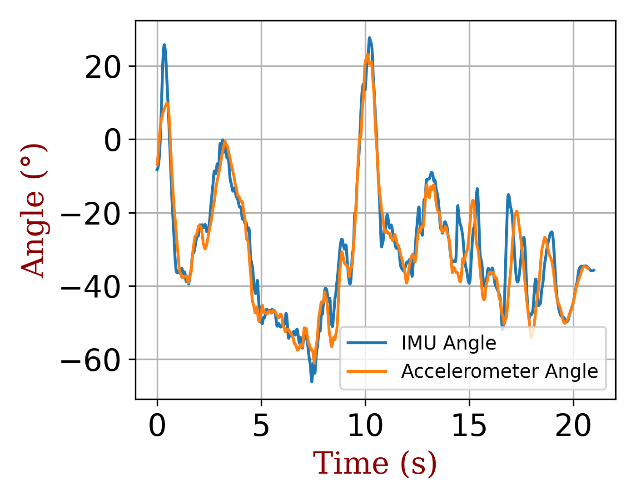  Fig S1.2 Reaching movement 2. RMSE: 5.9° |
| --- | --- |
| 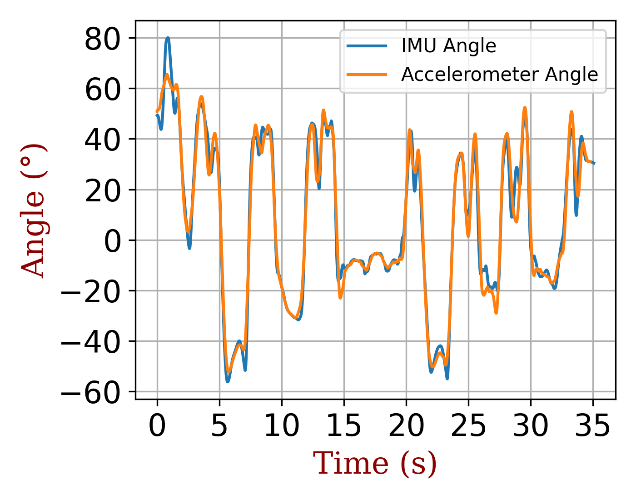  Fig S1.3 Movement: Put on a jacket. RMSE: 4.74° | 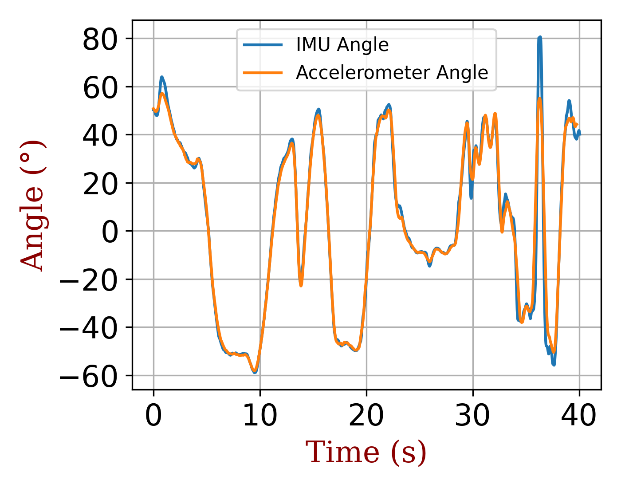  Fig S1.4 Movement: Removing a jacket. RMSE: 5.13° |

Figure S1: Comparison between IMU elevation angle (in blue) and accelerometric derived elevation angle (in orange) for four different upper limb movements. RMSE: Root Mean Square Error

In addition, we estimated the FuncUse-angular range of motion relationship from the IMU and accelerometer data on one subject during high and low intensity activities on 3 hours periods. The results are shown in Figure S2 and S3 respectively.

| 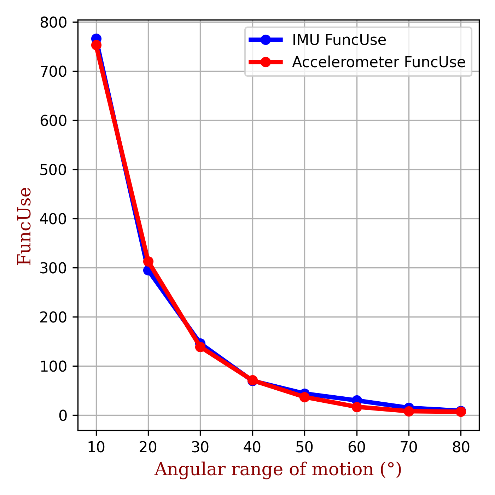  Figure S3.1 : Left side. RMSE: 10.08 movements | 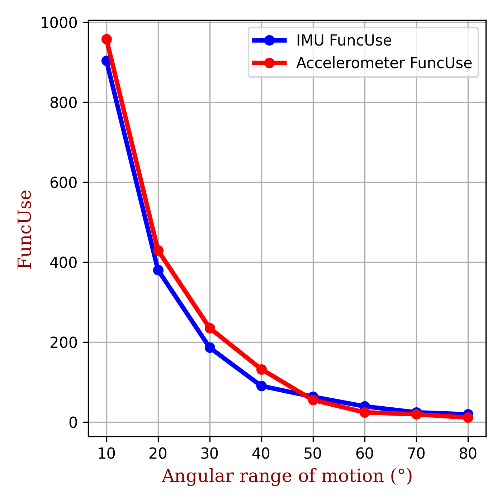  Figure S2.2 : Right side. RMSE: 35.1 movements |
| --- | --- |

Figure 2: FuncUse comparison between IMU and accelerometer data for “high intensity” activities.

| 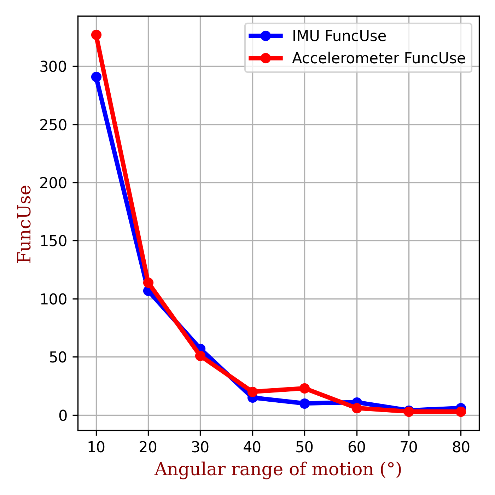  Figure S3.1: Left side. RMSE: 14.18 movements | 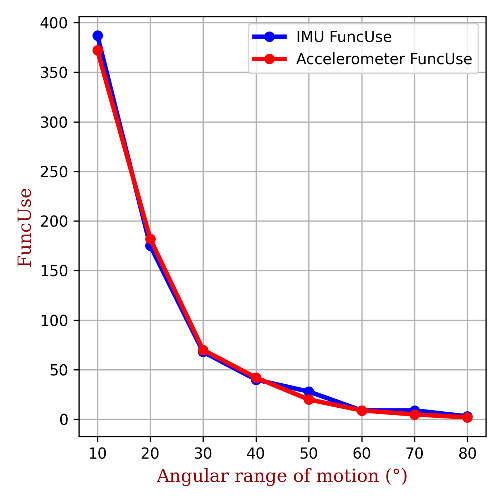  Figure S2.2: Right side. RMSE: 6.73 movements |
| --- | --- |

Figure 3: FuncUse comparison between IMU and accelerometer data for “low intensity” activities.

The results are qualitatively identical with a decrease in FuncUse as the angular amplitude of the movements increases. The RMSE of the FuncUse-angular range of motion relationship between gyroscopic and accelerometer data remains below 35 movements for high intensity activities and below 14 movements for low intensity activities. Finally, the estimation error of the FuncUse30 between the gyroscope and the accelerometer is at most 13% (FuncUse30: 421 movements with gyroscope vs 476 movements with accelerometer) for high intensity activities and at least 2.9% for low intensity activities (FuncUse30: 103 with gyroscope vs 106 with accelerometer).

Such results are considered acceptable for the use of Equation 1 for the estimation of the elevation angle and the FuncUse.
